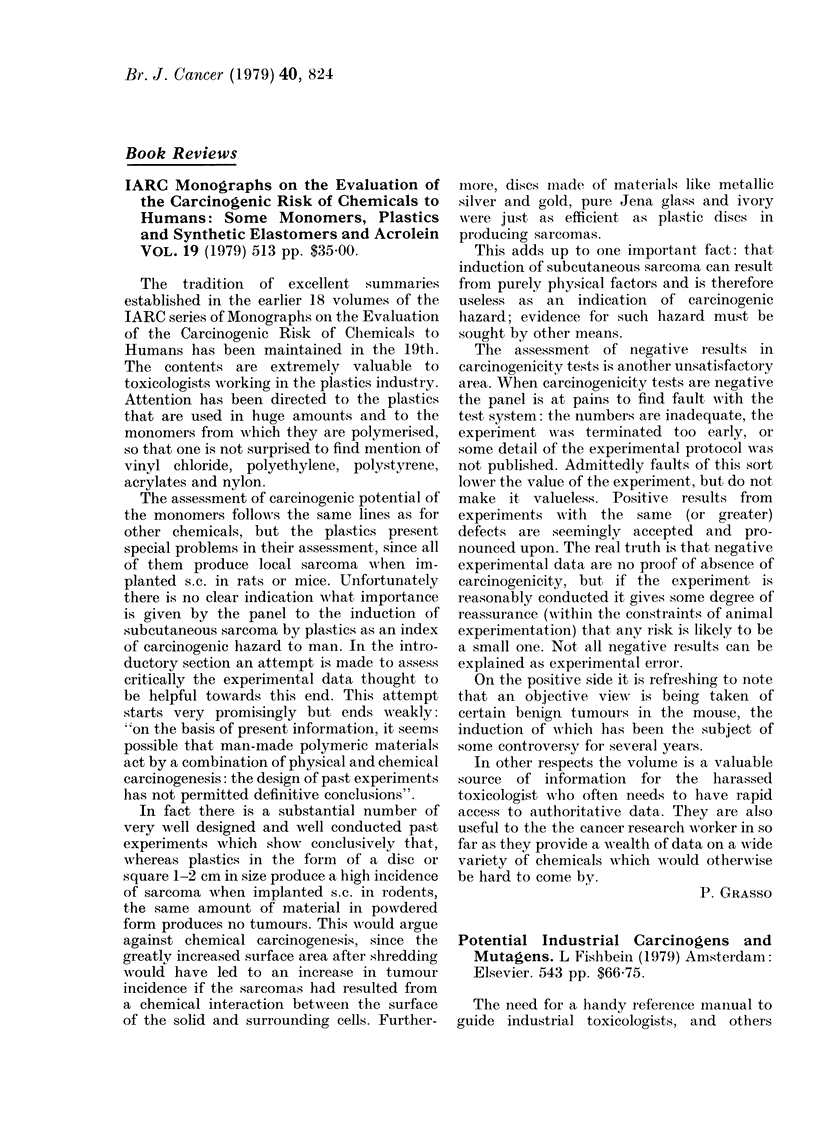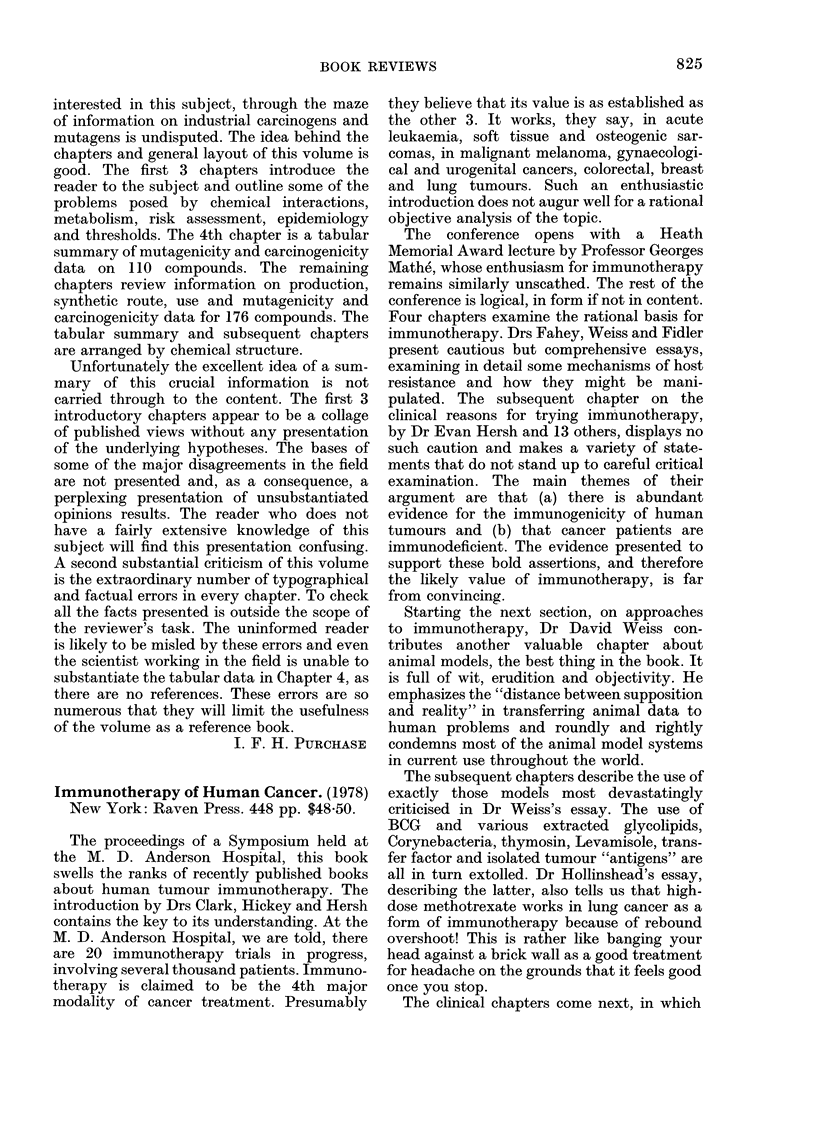# Potential Industrial Carcinogens and Mutagens

**Published:** 1979-11

**Authors:** I. F. H. Purchase


**Potential Industrial Carcinogens and Mutagens.** L Fishbein (1979) Amsterdam: Elsevier. 543 pp. $66·75.

The need for a handy reference manual to guide industrial toxicologists, and others interested in this subject, through the maze of information on industrial carcinogens and mutagens is undisputed. The idea behind the chapters and general layout of this volume is good. The first 3 chapters introduce the reader to the subject and outline some of the problems posed by chemical interactions, metabolism, risk assessment, epidemiology and thresholds. The 4th chapter is a tabular summary of mutagenicity and carcinogenicity data on 110 compounds. The remaining chapters review information on production, synthetic route, use and mutagenicity and carcinogenicity data for 176 compounds. The tabular summary and subsequent chapters are arranged by chemical structure.

Unfortunately the excellent idea of a summary of this crucial information is not carried through to the content. The first 3 introductory chapters appear to be a collage of published views without any presentation of the underlying hypotheses. The bases of some of the major disagreements in the field are not presented and, as a consequence, a perplexing presentation of unsubstantiated opinions results. The reader who does not have a fairly extensive knowledge of this subject will find this presentation confusing. A second substantial criticism of this volume is the extraordinary number of typographical and factual errors in every chapter. To check all the facts presented is outside the scope of the reviewer's task. The uninformed reader is likely to be misled by these errors and even the scientist working in the field is unable to substantiate the tabular data in Chapter 4, as there are no references. These errors are so numerous that they will limit the usefulness of the volume as a reference book.

I. F. H. PURCHASE